# Charting a Course for Smartphones and Wearables to Transform Population Health Research

**DOI:** 10.2196/42449

**Published:** 2023-02-07

**Authors:** William G Dixon, Sabine N van der Veer, Syed Mustafa Ali, Lynn Laidlaw, Richard J B Dobson, Cathie Sudlow, Tim Chico, Jacqueline A L MacArthur, Aiden Doherty

**Affiliations:** 1 Centre for Epidemiology Versus Arthritis Manchester Academic Health Science Centre University of Manchester Manchester United Kingdom; 2 Centre for Health Informatics Manchester Academic Health Science Centre Manchester United Kingdom; 3 Department of Biostatistics and Health Informatics King's College London London United Kingdom; 4 British Heart Foundation Data Science Centre Health Data Research UK London United Kingdom; 5 Big Data Institute Nuffield Department of Population Health University of Oxford Oxford United Kingdom

**Keywords:** mHealth, wearable, person-generated health data, population health research, devices, research, health, data, mobile health, clinical, digital

## Abstract

The use of data from smartphones and wearable devices has huge potential for population health research, given the high level of device ownership; the range of novel health-relevant data types available from consumer devices; and the frequency and duration with which data are, or could be, collected. Yet, the uptake and success of large-scale mobile health research in the last decade have not met this intensely promoted opportunity. We make the argument that digital person-generated health data are required and necessary to answer many top priority research questions, using illustrative examples taken from the James Lind Alliance Priority Setting Partnerships. We then summarize the findings from 2 UK initiatives that considered the challenges and possible solutions for what needs to be done and how such solutions can be implemented to realize the future opportunities of digital person-generated health data for clinically important population health research. Examples of important areas that must be addressed to advance the field include digital inequality and possible selection bias; easy access for researchers to the appropriate data collection tools, including how best to harmonize data items; analysis methodologies for time series data; patient and public involvement and engagement methods for optimizing recruitment, retention, and public trust; and methods for providing research participants with greater control over their data. There is also a major opportunity, provided through the linkage of digital person-generated health data to routinely collected data, to support novel population health research, bringing together clinician-reported and patient-reported measures. We recognize that well-conducted studies need a wide range of diverse challenges to be skillfully addressed in unison (eg, challenges regarding epidemiology, data science and biostatistics, psychometrics, behavioral and social science, software engineering, user interface design, information governance, data management, and patient and public involvement and engagement). Consequently, progress would be accelerated by the establishment of a new interdisciplinary community where all relevant and necessary skills are brought together to allow for excellence throughout the life cycle of a research study. This will require a partnership of diverse people, methods, and technologies. If done right, the synergy of such a partnership has the potential to transform many millions of people’s lives for the better.

## Introduction

Consumer digital devices provide a major opportunity to transform our understanding of the mechanisms, determinants, and consequences of diseases, including arthritis, dementia, and heart disease [1-3]. Most people in high- and low-income societies now own and regularly use consumer digital devices. Around 9 in 10 people own a smartphone in the United Kingdom [4], while one-fifth of US adults own wearable technologies, like smartwatches and fitness trackers [5]. This high level of device ownership means that many people could contribute to health research from the comfort of their home by offering small amounts of time to share data and help address clinical questions that matter to them.

Considering the wide range of the types of data available and the frequency and duration with which they are, or could be, collected, a significant step toward changes in how we conduct health research is within reach. Such data provide a much clearer picture of the daily rhythms of health, well-being, and disease, as well as the environment in which these occur. The touch screens, motion sensors, microphones, cameras, location sensors, and other technologies within these devices allow us to rethink how we measure things that are important and relevant to health research. Physical activity, for example, is an important risk factor for many diseases that is also negatively impacted when living with a condition, such as arthritis or stroke. Wrist-worn devices offer an opportunity to shift from the use of subjective questionnaires (eg, those asking “In a typical week, on how many days did you do 10 minutes or more of moderate physical activities like carrying light loads, cycling at normal pace?” [6]) to the continuous objective measurement of physical activity patterns [7]. One can easily see the differences in granularity, validity, reliability, and data collection burden between these two methods.

Smartphones and wearables have, however, not been used for research at scale beyond a handful of high-profile studies. Among the better examples of large-scale studies is the COVID Zoe study, which demonstrated that the mass collection of digital person-generated health data is both feasible and valuable, providing important early evidence for public health that anosmia is a key symptom of COVID-19 [8]. Further, a study on the Apple Watch (Apple Inc) proved that smartwatches can detect clinically meaningful heart rhythm patterns, like atrial fibrillation [9]. However, despite these studies illustrating digital devices’ major potential for answering important research questions at speed and scale, this opportunity has yet to be fully exploited. Furthermore, no large-scale study has yet established the linkage of longitudinal wearable data to major clinical outcomes. Such linkage is important, as it brings together key ingredients for important population health research questions; for example, it would allow us to understand whether digital interventions for improving physical activity result in improvements in hard clinical outcomes, like a reduction in myocardial infarctions or a reduction in the number of people who develop diabetes.

In this viewpoint paper, we make the case that there remains a critical need to collect and link digital person-generated health data at scale by illustrating that such data are *required and necessary* to answer many vital research questions that matter to patients, clinicians, and policy makers, and we describe the requirements for collecting and linking such data. We then summarize what is needed to advance progress in this important and emerging field.

## Opportunities

To illustrate the importance of and need for digital person-generated health data, we reviewed priority research questions for a number of common conditions. The James Lind Alliance is a UK initiative that brings together patients, carers, clinicians, and researchers in priority setting partnerships to identify and prioritize the top 10 most important unanswered questions or uncertainties for a given disease area [10]. Although there are other means for identifying research priorities, the James Lind Alliance follows a standardized process that is common across diseases, plus it brings together the views of different stakeholders. We reviewed the lists of the top 10 questions for the following six common disorder areas: arthritis, diabetes, chronic obstructive pulmonary disease, inflammatory bowel disease, stroke, and mental health. Each disorder area contained at least one question (often several questions) that would be optimally addressed with digital person-generated health data, with or without additional linked clinical data. Textbox 1 contains some of these questions, showing the need to collect data on physical and mental health symptoms and environmental factors, such as diet and exercise.

**Examples of as yet unanswered questions that digital person-generated health data would optimally address as part of the solution. These questions come from James Lind Alliance Priority Setting Partnerships exercises for 6 common disorder areas.**

**Example questions**

1. “Is regular exercise and physical activity effective at reducing disease progression [in hip and knee osteoarthritis]?” [11]
2. “How do stress and anxiety influence the management of type 2 diabetes and does a positive mental wellbeing have an effect?” [12]
3. “What is the best way to tell the start of an exacerbation [of chronic obstructive pulmonary disease] from day-to day variation in symptoms?” [13]
4. “What role does diet have in the management of mildly active or inactive ulcerative colitis or Crohn’s Disease to achieve normal daily activities and symptom control?” [14]
5. “How common are psychological problems and what impact do they have on the lives of people affected by stroke?” [15]
6. “How do certain mental health conditions (e.g. depression) affect how people engage with technology?” [16]

A recent review of what happens after a priority setting exercise [17] noted that addressing a priority topic requires researchers to design a dedicated study. The opportunity to collect data directly from patients at scale via digital devices could now help researchers and the public to address many top priority questions more easily and robustly. However, before we can harness this potential, we need to chart a course to overcome the barriers to conducting such large-scale population health research well.

We ran 2 parallel and complementary initiatives in 2021 to investigate possible solutions for successfully using smartphones and wearable data in population health research. The first was a British Heart Foundation Data Science Centre workshop, which focused on wearables for cardiovascular research [18]. The second was a roundtable event that considered the future of digital person-generated health data for UK health research and was hosted by the Centre for Epidemiology Versus Arthritis [19]. Both initiatives brought together multiple stakeholders, including patients, health care professionals, researchers, funders, policy makers, governance experts, and industry representatives, reflecting the importance of widespread consultation. The reports on these two initiatives [18,19] underline the major opportunities for population health research using digital person-generated health data. They both also recognize that countries such as the United Kingdom are in a particularly strong position, given the possibility of linking person-generated health data with routinely collected health data, such as those from the National Health Service, which has universal access to health care and cradle-to-grave health records. There is a pressing need for national-scale studies in which large numbers of smartphone and wearable users are invited to consent to the sharing of their device data to allow these data to be linked to their routinely collected health care information for research. These mobile data could enhance population health research if they could be integrated into emergent digital infrastructure to support health data research using routinely collected electronic health record data [20] and into large population cohort studies with genetic and deep phenotypic information, such as information from UK Biobank [21] and Our Future Health [22].

## Requirements

Well-conducted population health research must consider potential challenges during study design and how to navigate them—a key area of discussion in both aforementioned initiatives. The recruitment of study participants based on device ownership would be skewed, as not everyone owns a device, introducing possible selection bias; for example, people who use wearable activity trackers are more active, younger, and more affluent than those who do not [23]. Study results must be useful and ideally generalizable to a wider population. It is vital that research does not worsen already existing health, social, and racial inequalities [24]. Researchers need to be able to set up studies easily and efficiently, use high-quality study designs, and have access to the right data collection tools that are both stable and flexible [25]. Data harmonization and interoperability are important challenges; the proliferation of devices with different proprietary software algorithms for determining measures like step count has resulted in researchers being unable to trust the outputs of consumer devices. Different devices provide different step counts for the same activity and vary greatly in accuracy [26]. There is a need to generate reproducible digital phenotypes from raw sensor data and low-level features (eg, measures of mobility or sleep), as well as the need to understand the environment and context in which data are generated, which may need more qualitative approaches. There is also a need for the harmonization of self-reported information, such as symptoms within and across diseases, especially as the number of people with multiple long-term conditions increases [27]. Public trust, engagement, and involvement are essential from the earliest point. These involve defining and prioritizing the most important, relevant, and feasible questions to address; designing the most appropriate studies; co-designing user-friendly devices and apps [28]; inviting people to join a study through the remote consent process [29]; and keeping them motivated to optimize ongoing engagement [30]. It is also important to enable participants to maintain and feel in control of where and how their data are used and to share the benefits and results of their contributions [31].

## Proposed Solutions

Realizing the potential of patient-generated data in health care research requires a new interdisciplinary community to be established. Academics from diverse areas, such as epidemiology, software development, data science and biostatistics, psychometrics, and behavioral and social science, need to work with patients and health care professionals, alongside colleagues from industry who could contribute skills such as hardware and software engineering, user interface design, cybersecurity, and data management. Only by operating across disciplinary boundaries can we develop the foundations for future high-quality research and in turn support a wider group of interested, but so far relatively inexperienced, researchers. This can be done by defining and supporting best practices and providing access to the tools and methods needed to address the highest priority questions.

In countries such as the United Kingdom, a crucial requirement is understanding how we can best link digital person-generated health data with national health care data sets for research in a way that is understandable, feasible, and acceptable to participants and provides them with the option of retaining control over how and by whom their data are used. This linkage should use existing national infrastructure, such as trustworthy research environments [32]. In addition to the technical infrastructure, such linkage also requires the development and evaluation of a range of approaches and methods, such as determining how best to recruit and remotely consent participants, securely storing and linking the different data types across different geographical areas, ensuring the validity and harmonization of data across devices, engaging participants through feedback, and providing them with control to ensure that we maintain trust. In this context, the prominent involvement of patients and the public is the most vital factor as we proceed; we can only undertake large-scale population health research if people are willing to participate in and consent to the collection and sharing of their data repeatedly over time. Before asking this of patients and the public, we must ensure that research is done in a way that is acceptable, is valuable, and has meaning and relevance to them [33,34].

We believe that the time is right to create the partnerships, platforms, tools, and methods that will allow us to collect data directly from patients via digital devices; securely link these data to their routinely collected health care data in a trustworthy way; and answer many more questions that matter to patients, health care professionals, policy makers, and the wider public.
